# Modern contraceptive use among unmarried girls aged 15–19 years in South Western Nigeria: results from a cross-sectional baseline survey for the Adolescent 360 (A360) impact evaluation

**DOI:** 10.1186/s12978-020-01056-w

**Published:** 2021-01-06

**Authors:** Emily E. Crawford, Christina J. Atchison, Yewande P. Ajayi, Aoife M. Doyle

**Affiliations:** 1 Binomial Optimus Limited, Blue Hill, PLOT 538 Natasha Akpoti Street Kado, Abuja, FCT Nigeria; 2 Imperial College London, School of Public Health, St Mary’s Hospital, Norfolk Place, London, W2 1PG UK; 3 MRC International Statistics and Epidemiology Group, London School of Hygiene and Tropical Medicine, Keppel St, Bloomsbury, London, WC1E 7HT UK

**Keywords:** Adolescent health, Reproductive health, Modern contraception, Nigeria, Sub-Saharan Africa, Impact evaluation

## Abstract

**Purpose:**

Adolescents 360 (A360) is an initiative being rolled out across Nigeria with the aim of increasing voluntary modern contraception use among women aged 15 to 19 years. Using evaluation study baseline data, we identified sexuality, fertility and contraceptive use characteristics of young unmarried girls in South Western Nigeria.

**Methods:**

A cross-sectional baseline survey of unmarried girls aged 15 to 19 years was conducted in Ogun state, Nigeria in August 2017. A clustered sampling design was used. We identified determinants of modern contraceptive use in this subpopulation using logistic regression.

**Results:**

Of 12,024 women interviewed, 15.3% reported sexual intercourse in the past year. The majority of respondents (79.6%, 9525/11,967) had heard of contraception. 45.3% of sexually active respondents were using a modern contraceptive method. Of those using any method of contraception, male condoms (50.3%) were the most widely used modern method followed by the emergency contraceptive pill (16.7%). Following adjustment for socio-demographic characteristics, there was evidence that the use of modern contraception was positively associated with having never given birth, living in an urban area, current enrolment in education, high level of education, high socioeconomic status, exposure to information about contraception, perceived social support for contraception, and self-efficacy for contraception.

**Conclusions:**

In South Western Nigeria, unmarried sexually active adolescent girls have relatively low levels of modern contraceptive use. Programmes should aim to increase access to modern contraception and to increase social support and acceptability of contraceptive use.

## Plain English summary

Purpose: Many sexually active young women who do not want to get pregnant are not using modern contraception to prevent pregnancy. Adolescents 360 (A360) hopes to help young Nigerian women prevent pregnancy if they want to. This paper explains which young women in South West Nigeria are more likely to use modern contraception than others.

Methods: We interviewed unmarried girls 15–19 years old in Ogun State, Nigeria. We asked them about their sexual activity and attitudes towards contraception.

Results: We interviewed 12,053 women. 15.3% said they had sex in the past year. Most women had heard of contraception. Even though they had heard about modern contraception, they did not know any place where they would feel comfortable getting it. Less than half of the women who had sex were using modern contraception. Among the women who were using modern contraception, half (50%) were using male condoms followed by the emergency contraceptive pill (17%). Women were more likely to use modern contraception if they had never given birth before, lived in a city, had spent more time in school, were in school now, had heard about contraception, felt their family and friends supported them in using contraception, and felt able to get contraception.

Conclusions: Programs wanting to help young unmarried girls in Nigeria to prevent pregnancy should make available safe places where they can get contraception. These programs should also work with friends and families of girls to help these women feel more supported to get contraception.

## Introduction

Despite increases in both knowledge about modern contraception (MC) and desire to delay or space child births, many sub-Saharan African (SSA) women still have low uptake of modern contraceptives and high rates of unmet need [1, 2]. Closing this gap is a global priority currently being addressed through the Family planning 2020 (FP2020) initiative and United Nation’s Sustainable Development Goals (SDG) 3 and 5 focusing on health and well-being for all and gender equality [3, 4]. The gap in access to MC is particularly pronounced for girls in LMICs as compared to high-income countries; for instance, 22% of girls aged 15–19 years in Nigeria report using a MC at the last time they had sexual intercourse, compared with more than half in the UK and 39% in the USA [5–7]. Low MC uptake contributes to high adolescent pregnancy rates, and poor health outcomes including maternal morbidity and mortality, and neonatal and under-five child mortality [1, 8, 9]. In addition, unplanned pregnancy can lead to other severe social and economic consequences for girls, their families, and society as a whole, including not reaching their potential for educational achievement, and not getting a paid job, often leading into a cycle of poverty [8, 10].

Most studies to date have focused on the factors associated with use of MC by women of reproductive age (15–49 years) [1], with adolescents (15–19 years) usually underrepresented [8]. Most literature on access to MC has focused on married women, though unmet demand for and access to MC among adolescents are known to differ with women’s marital status, with unmet need for MC highest among unmarried girls [11, 12]. For the goals of FP2020 and SDG 3 and 5 to be achieved, more information is needed on factors associated with contraceptive use among unmarried girls in low contraceptive prevalence settings such as Nigeria.

While studies on contraceptive use among adolescents in Africa are scarce, studies on determinants of adolescent pregnancy can shed light on factors that may be associated with contraceptive use. Systematic reviews have shown poverty, education, parental involvement, access to education on contraceptives, and misconceptions about contraception to be associated with adolescent pregnancy in African countries [13, 14]. Nigerian adolescents face unique barriers in obtaining knowledge of and access to contraception. Although Nigeria adopted a curriculum for Family Life and HIV Education in 2003; this curriculum mentions safer sex in the context of HIV prevention, but omits any reference to contraception [15–17]. Access to contraception can be more challenging for adolescents; while age of consent for sexual activity in Nigeria is 18 [18], access to contraceptives is not officially restricted based on age [19]. Many providers, however, restrict access to contraception based on age or parity restrictions of their own; this is likely an act of self-preservation in the face of unclear laws [20].

Adolescents 360 (A360) is an initiative being rolled out across Ethiopia, Nigeria and Tanzania to increase voluntary uptake of MC among sexually active women aged 15–19 years [21]. A360 is country-adapted, and in Southern Nigeria combines community advocacy alongside safe spaces for girls to learn about contraception and to access MC methods [21]. Funding for A360 comes from the Bill and Melinda Gates Foundation and the Children’s Investment Fund Foundation.

Using baseline survey data collected as part of the A360 programme evaluation, we describe sociodemographic characteristics, sexual activity, and contraceptive use of unmarried girls aged 15–19 years in Ogun State, South Western Nigeria, and determined factors associated with MC use among unmarried sexually active girls.

## Methods

Between August and October 2017, we conducted a cross-sectional household survey among unmarried girls aged 15–19 years in Shagamu and Ado-Odo Ota local government areas (LGA) in Ogun State, South Western Nigeria. These LGA were selected based on planned implementation of the A360 Initiative in Ado-Odo Ota, with Shagamu selected as a comparison. Ogun State has an estimated total population of 5.8 million [22], and shares its Southern border with Lagos state. Overall, 36% of the female household population has no education, and the median age at first marriage for women is 19.1 years [7].

Full details of the sampling strategy and sample size calculations are described elsewhere [21].

Girls were included if they were 15–19 years old, unmarried, and living in the study sites at the time of the survey. Respondents were classified as unmarried if they reported that they did not have a husband or were not living as married with a cohabiting male partner.

A cluster sampling design was used. The primary sampling unit was the 2006 census enumeration area (EA), the smallest administrative unit of Nigeria. All eligible consenting unmarried girls aged 15–19 years residing in households in these EAs were invited to be interviewed [21]. If potentially eligible participants were not available, two revisits were made to attempt to hold interviews. At the 2006 census, each EA was defined to have approximately 100 households, however, the number of households per EA in 2017 was likely to vary. We took a simple random sample of EAs across the two LGAs and, to maximise the efficiency of the survey logistics, sampled clusters of approximately 100 households in or near the sampled EAs. Thus, if an EA contained fewer than 100 households, we randomly sampled a neighbouring EAs and enumerated all households in that EA as well. If an EA exceeded 150 households, we divided the EA into approximately equal sections of 75–100 households and randomly selected one section for enumeration. A simple random sample of 716 EAs was needed to reach the target sample size of 12,000 women.

The questionnaire was adapted from research instruments used in the Nigeria Demographic and Health Survey (DHS) [23] and FP2020 survey [3]. Questionnaires were administered face-to-face by female interviewers aged between 18 and 26 years using pre-programmed tablet computers [21]. Interviews were conducted in a private location in the respondent’s homes in August and September 2017. The questionnaire had three components: (1) sociodemographic characteristics (2) fertility characteristics and preferences, and (3) contraceptive knowledge, attitudes and practices.

Only respondents who reported sexual intercourse in the last 12 months were considered sexually active hence asked questions about contraceptive use [21].

Participants voluntarily provided verbal informed consent. A waiver of written consent was granted given the sensitive nature of the topics discussed. Women aged 15–17 years provided verbal assent and verbal informed consent was also sought from her parent/guardian. The study protocol was approved by the National Health Research Ethics Committee of Nigeria (Ref: NHREC/01/01/2007–25/05/2017) and the London School of Hygiene and Tropical Medicine Ethics Committee (Ref: 14145).

### Study outcome

The Modern Contraceptive Prevalence Rate (mCPR) among unmarried-sexually active girls aged 15–19 years was defined as follows:

$$mCPR=\frac{\begin{array}{c}Number\, of\,unmarried\,sexually\,active\,*\,girls\,aged\,15-19\,years\,reporting \\ use\,of\,modern\,contraceptives\,at\,the\,time\,of\,the\,survey \end{array}}{Number\,of\,unmarried\,sexually\,active\,*\,girls\,aged\,15-19\,years}$$

*Self-reported that they were sexually active in the 12 months prior to the survey.

Modern contraception was defined to include the following: male and female sterilisation, contraceptive implants, intrauterine contraceptive devices, injectables, contraceptive pill/oral contraceptives, emergency contraceptive pill, male condom, female condom, standard days method (SDM), lactational amenorrhoea method, diaphragm, spermicides, foams and jelly [24].

### Statistical analysis

Data analysis was conducted in Stata V.15.

We used robust SEs to account for the clustered sampling design. We described socio-demographic characteristics, sexual activity, and contraceptive use of unmarried girls. Logistic regression was performed for the subpopulation of sexually active girls. We obtained odds ratios (ORs) for the association of each explanatory variable with use of MC. Wald tests adjusted for the clustered sampling design were used at each step of the analysis. Variables with p-value of < 0.2 in bivariate analysis were included in multivariable regression models. The association between mCPR and age was not adjusted for other explanatory variables. The associations between mCPR and other sociodemographic variables were adjusted for age, an a priori potential confounder. For the remaining explanatory variables, models were adjusted for age and sociodemographic factors which had age-adjusted p-values of < 0.2. Variables with p value < 0.05 in the adjusted analysis were considered to have some evidence of an association with mCPR. This strategy allowed us to assess the effect of variables adjusted for distal a priori potential confounders.

Socioeconomic position was created from a series of questions about household items, dwelling materials and access to a bank account using the ‘Nigeria Equity Tool’. This tool uses different weights attached to each answer to create a composite score which was then split into quintiles according to the national thresholds [25]. Variables for contraception knowledge, holding misconceptions and self-efficacy were created as scores from 0 to 5 for knowledge, and 0 to 4 for holding misconceptions and self-efficacy based on the overall score for each individual statement in each category. A score of 1 was given if the respondent agreed with the statement and 0 if she disagreed or answered ‘don’t know’. A maximum score of 5 for knowledge and 4 for self-efficacy would indicate that the respondent correctly agreed with all five knowledge statements and felt able to achieve all four self-efficacy behaviours. A maximum score of 4 for holding misconceptions would be interpreted as believing all four myth statements about contraception. As per DHS definition, this study considered three outcomes for unmet need for MC: total unmet need, unmet need for spacing, and for limiting [7]. The total unmet need was the sum of unmet need for spacing and limiting.

## Results

In total 13,481 potentially eligible unmarried girls (15–19 years) were identified with 12,053 interviewed and 12,024 (89.2%) interviewed and responding to sexual history questions. 1428 girls (10.5%) were identified in the household listing but not reachable or available to be interviewed.

### Sexual activity among respondents

Overall, 15.3% (1844/12,024) of adolescent women had been sexually active during the 12 months preceding the survey. The median age was 18 years (IQR 18–19) for sexually active and 17 years (IQR 15–18) for non-sexually active women (Table 1). The highest level of education achieved by the majority of respondents was secondary level education. Only 30.5% of sexually-active adolescent women were currently in education compared to 62.2% of non-sexually active women (Table 1).

**Table 1:** Characteristics of unmarried sexually active and non-sexually active women aged 15–19 years in Ogun, South Western Nigeria^a^

Characteristic	Sexually active, N = 1844	Non-sexually active, N = 10,180	p value^b^
Sociodemographic factors	n (%)^a^	n (%)^a^	
Age (years)			
15	66 (3.6)	3213 (31.6)	
16	119 (6.5)	1854 (18.2)	
17	233 (12.6)	1692 (16.6)	
18	552 (29.9)	2006 (19.7)	
19	874 (47.4)	1415 (13.9)	< 0.001
Age (years)^c^	18 (18–19)	17 (15–18)	
Religion			
Catholic	36 (2.0)	245 (2.4)	
Protestant/other christian	1181 (64.1)	6195 (60.9)	
Muslim	620 (33.6)	3703 (36.4)	
Traditional	7 (0.38)	31 (0.30)	
No religion	0	1 (0.01)	
Other	0	3 (0.03)	0.15
Highest education level achieved			
No education	21 (1.1)	125 (1.2)	
Quranic only^d^	0	4 (0.04)	
Primary	109 (5.9)	363 (3.6)	
Secondary	1547 (83.9)	9301 (91.4)	
Higher education	167 (9.1)	386 (3.8)	< 0.001
Currently in education			
Yes	563 (30.5)	6328 (62.2)	
No	1281 (69.5)	3847 (37.8)	< 0.001
Type of residence			
Urban	662 (35.9)	3621 (35.6)	
Semi-urban	756 (41.0)	4257 (41.8)	
Rural	426 (23.1)	2302 (22.6)	0.87
Socioeconomic level			
Lowest quintile	6 (0.33)	15 (0.15)	
Second lowest quintile	6 (0.33)	44 (0.44)	
Middle quintile	62 (3.4)	213 (2.1)	
Second highest quintile	355 (19.7)	1587 (16.0)	
Highest quintile	1376 (76.2)	8060 (81.3)	< 0.001
Exposure to information about contraception			
Ever heard about contraception			
Yes	1664 (90.7)	7861 (77.6)	
No	171 (9.3)	2271 (22.4)	< 0.001
Heard about contraception in the media in last 12 months?			
Yes	398 (23.9)	2177 (27.7)	
No	1266 (76.1)	5684 (72.3)	0.002
Heard about contraception from health sector sources in last 12 months?			
Yes	414 (24.9)	1103 (14.0)	
No	1250 (75.1)	6758 (86.0)	< 0.001
Heard about contraception from interpersonal sources in last 12 months?			
Yes	986 (59.3)	3941 (50.1)	
No	678 (40.8)	3920 (49.9)	< 0.001
Knows a place where or a person from whom she would feel comfortable accessing contraception?^e^			
Yes	672 (40.5)		
No	923 (55.7)		
Don’t know	63 (3.8)		
Social networks			
Perception that partner supports her using contraception^e^			
Yes	1058 (65.0)		
No	354 (21.7)		
Don’t know	216 (13.3)		
Perception that mother supports her using contraception^e^			
Yes	383 (25.7)		
No	747 (50.2)		
Don’t know	359 (24.1)		
Perception that friends supports her using contraception^e^			
Yes	925 (55.6)		
No	367 (22.1)		
Don’t know	372 (22.4)		
Individual knowledge, attitudes and behaviours			
Knowledge about contraception^f^			
0–1	60 (3.6)	547 (7.0)	
2–3	334 (20.1)	2126 (27.0)	
4–5	1270 (76.3)	5188 (66.0)	< 0.001
Misconceptions about contraception^g^			
0–1	526 (31.6)	2642 (33.6)	
2–3	851 (51.1)	4043 (51.4)	
4	287 (17.3)	1176 (15.0)	0.06
Self-efficacy for contraception^e,h^			
0–1	280 (17.1)		
2–3	609 (37.3)		
4	745 (45.6)		
Timing of most recent sexual activity			
Within past week	302 (16.4)	0	
Within past month	582 (31.6)	0	
Within past year	960 (52.1)	0	
More than 1 year ago	0	1199 (11.8)	
Never	0	8981 (88.2)	< 0.001
Age at first sexual intercourse			
< 15	192 (10.6)	257 (24.7)	
15–17	1103 (61.3)	688 (61.9)	
18–19	504 (28.0)	148 (13.3)	
Age (years)^c^	17 (15–18)	16 (15–17)	< 0.001
Ever been pregnant			
Yes, currently pregnant	98 (20.3)	0	
Yes, currently not pregnant	379 (78.5)	166 (98.8)	
Yes, not sure if currently pregnant	6 (1.2)	2 (2.2)	
No	1358 (73.6)	10,009 (98.3)	
Don’t know	3 (0.16)	3 (0.03)	< 0.001
Ever given birth			
Yes	178 (9.7)	99 (0.97)	
No	1666 (90.4)	10,081 (99.0)	< 0.001
Unmet need for modern contraception^e,i^			
No unmet need	1223 (71.0)		
Unmet need for spacing	496 (28.8)		
Unmet need for limiting	3 (0.17)		
Total unmet need	499 (29.0)		

^a^The figures refer to N (%)

^b^p value obtained using chi-squared test

^c^Median (IQR)

^d^In Nigeria, apart from the formal educational system, a non-formal Arabic and Islamic Educational System operates among the Nigerian Muslims, through Quranic schools

^e^Only respondents who reported sexual intercourse in the last 12 months were asked these questions

^f^Scored based on the responses to the following five questions: (1) preventing unwanted pregnancies is a benefit of contraception, (2) some contraceptive methods reduce sexually transmitted infections, (3) modern contraception can help with delaying having a first child, (4) modern contraception can help with child spacing and (5) using modern contraception can allow a woman to complete her education, find a better job and have a better life

^g^Scored based on the responses to the following four questions: (1) some modern contraception can stop an adolescent woman from ever being pregnant again even after she stops using it, (2) if a modern contraception changes an adolescent woman’s menstrual bleeding, it’s bad for her health and can harm her womb, (3) some modern contraceptives can make girls permanently fat and (4) girls who use modern contraception sleep with too many men

^h^Scored based on the responses to the following four questions: (1) felt able to start a conversation with her partner about contraception, (2) felt able to use a method of contraception even if her partner did not want her to, (3) felt able to obtain information on contraception services and products if she needed to and (4) felt able to obtain a contraceptive method if she decided to use one

^i^DHS definition [43]. Women reporting mistimed or unwanted pregnancies or recent births were considered having unmet need for spacing and for limiting, respectively [43]. In addition, fecund women who wanted children 2 or more years in the future, or were undecided whether/when they wanted a child were regarded as having an unmet need for spacing. Fecund women who wanted no more children were regarded as having an unmet need for limiting [43]. The denominator for the calculation of unmet need is the total of unmarried women aged 15–19 years. The numerator includes only women who were not using contraception at the time of the survey [7]

The majority of respondents (sexually active: 90.7%, non-sexually active: 77.6%; p < 0.001) had heard of contraception. Sexually-active respondents were more likely to have good knowledge (score 4–5) about contraception than non-sexually active respondents (76.3% vs. 66.0%; p < 0.001). A similar proportion held two or more misconceptions (score ≥ 2) about contraception (sexually-active: 68.4%, non-sexually active: 66.4%; p 0.06) (Table 1). Over half of sexually active adolescent women felt supported by their partners and friends to use contraception, compared to only about a quarter who felt that they had their mother’s support. In addition, only 40.5% of sexually active women knew of a place where or a person from whom they would feel comfortable accessing contraception. In total, 45.6% had the maximum self-efficacy score of 4, with similar proportions (55–59%) responding positively to each of the individual self-efficacy statements.

The majority of adolescent women reporting no sexual activity within the last 12 months had never had sexual intercourse and 11.8% reported sexual activity more than a year ago. Of note, the median age of first sexual activity in these women was lower than those reporting sexual activity within the last 12 months (age: 16 years vs. 17 years). About a quarter of sexually active women had been pregnant and 1 in 10 had given birth. Excluding women who were currently pregnant, half of those who reported having ever been pregnant also reported never having given birth (276/553 (49.9%)).

Unmet need for MC was 29.0%, made up almost entirely of unmet need for spacing (Table 1).

### Contraceptive use among sexually active respondents

Overall, 63.2% (1165/1844) of unmarried sexually active respondents were using any contraceptive method, with 45.3% (836/1844) using a modern method, and 17.8% (329/1844) using traditional contraceptive methods (Table 2). Male condoms were the most widely used modern method, used by 50.3% (586/1165) of those using any method. The emergency contraceptive pill was the next most popular modern method, used by 16.7% (194/1165) of contraceptive users. Traditional methods of contraception were commonly used with 20.7% (241/1165) of contraceptive users reporting use of the withdrawal method.

**Table 2:** Contraception use by unmarried sexually active women aged 15–19 years in Ogun, South Western Nigeria (%, (95% CI))

Characteristic	Number (N = 1844)^a^	% (95% CI)
Any method	1165	63.2 (60.6–65.7)
Any modern method^b^	836	45.3 (42.9–47.8)
Any traditional method	329	17.8 (15.9–19.9)
Not currently using	572	31.0 (28.7–33.4)
Don’t know	8	0.43 (0.20–0.94)
No response	99	5.4 (4.5–6.5)
Total	1844	100.0
Method	N = 1165	
Modern method		
Female sterilization	1	0.09 (0.01–0.61)
Implant	8	0.69 (0.35–1.4)
IUCD	1	0.09 (0.01–0.61)
Injectables	5	0.43 (0.18–1.0)
Oral contraceptive pill	26	2.2 (1.5–3.3)
Emergency pill	194	16.7 (14.6–19.0)
Male condom	586	50.3 (47.4–53.3)
Female condom	4	0.34 (0.13–0.91)
SDM	7	0.84 (0.40–75.8)
LAM	4	0.34 (0.13–0.91)
Traditional method		
Rhythm method	5	0.43 (0.15–1.2)
Withdrawal	241	20.7 (18.2–23.5)
Drinks salt solution	46	3.9 (3.0–5.3)
Other	37	3.2 (2.2–4.5)
Total	1165	100.0

Figures are % (95% CI)

^a^Unmarried girls who report sexually activity in last 12 months

^b^Modern methods include female sterilisation, male sterilisation, contraceptive pill (oral contraceptives), IUCD (intrauterine contraceptive devices), injectables (Depo-Provera), implants (Norplant), female condom, male condom, diaphragm, contraceptive foam and contraceptive jelly, LAM (lactational amenorrhoea method), SDM (standard days method), cycle beads

### Characteristics associated with use of MC

Among sexually active adolescent women, older age, higher level of educational attainment, being currently enrolled in school, living in an urban area, and higher socioeconomic position were all positively associated with use of MC methods (p-value < 0.05) (Table 3). After adjusting for sociodemographic variables, the odds of using MC were significantly lower for women who had not heard about contraception in the last 12 months in the media (adjOR 0.65, 95% CI 0.51–0.82) or from a health sector source (adjOR 0.53, 95% CI 0.42–0.68) compared with those that had heard this information. Those who did not know of a place or person from whom they would feel comfortable accessing contraception had lower odds of using MC compared with those who did (adjOR 0.65, 95% CI 0.53–0.81). The odds of using MC were lower for women who perceived that their partners did not support their use of contraception (adjOR 0.27, 95% CI 0.20–0.35) and for those who perceived lack of support from their friends (adjOR 0.54, 95% CI 0.42–0.70) compared with those who perceived such support. The odds of using MC increased with higher self-efficacy for contraception (score of 4 vs. score of 0–1; adjOR 3.1, 95% CI 2.3–4.2). The odds of using MC were significantly higher in women who had never given birth compared with those who had (adjOR 1.5, 95% CI 1.1–2.1).

**Table 3:** Factors associated with modern contraception use among unmarried sexually active women aged 15–19 years in Ogun, South Western Nigeria, (N = 1737)

Exposure category	No	Prevalence, n (%)	Unadjusted OR (95% CI)	p value	Adjusted OR (95% CI)	p value
Sociodemographic factors						
Age (years)						
15	60	20 (33.3)				
16	113	48 (42.5)				
17	221	94 (42.5)				
18	522	285 (54.6)				
19	821	389 (47.4)				
Per year increase			1.10 (1.00–1.20)	0.04		
Religion						
Catholic	34	18 (52.9)	1			
Protestant/other christian	1119	553 (49.4)	0.87 (0.47–1.6)			
Muslim	578	264 (45.7)	0.75 (0.40–1.4)			
Traditional	6	1 (16.7)	0.18 (0.02–1.7)	0.22		
Highest education level achieved^a^						
No education/primary	115	32 (27.8)	1		1	
Secondary	1459	698 (47.8)	2.4 (1.6–3.6)		2.4 (1.5–3.6)	
Higher education	163	106 (65.0)	4.8 (2.8–8.2)	< 0.001	4.6 (2.7–8.0)	< 0.001
Currently in education^a^						
Yes	549	284 (51.7)	1		1	
No	1188	552 (46.5)	0.81 (0.66–0.99)	0.04	0.73 (0.59–0.90)	0.003
Type of residence^a^						
Urban	630	334 (53.0)	1		1	
Semi-urban	719	349 (48.5)	0.84 (0.67–1.0)		0.84 (0.67–1.0)	
Rural	388	153 (39.4)	0.58 (0.44–0.76)	< 0.001	0.59 (0.45–0.77)	< 0.001
Socioeconomic level^a^						
Lowest to middle quintile	66	18 (27.3)	1		1	
Second highest quintile	334	144 (43.1)	2.0 (1.1–3.8)		2.0 (1.1–3.8)	
Highest quintile	1301	664 (51.0)	2.8 (1.5–5.1)	< 0.001	2.7 (1.5–5.0)	< 0.001
Exposure to information about contraception						
Heard about contraception in the media in last 12 months?^b^						
Yes	380	227 (59.7)	1		1	
No	1197	577 (48.2)	0.63 (0.50–0.79)	< 0.001	0.65 (0.51–0.82)	< 0.001
Heard about contraception from health sector sources in last 12 months?^b^						
Yes	394	248 (62.9)	1		1	
No	1183	556 (47.0)	0.52 (0.41–0.67)	< 0.001	0.53 (0.42–0.68)	< 0.001
Heard about contraception from interpersonal sources in last 12 months?^b^						
Yes	946	496 (52.4)	1		1	
No	631	308 (48.8)	0.87 (0.70–1.1)	0.18	0.88 (0.71–1.1)	0.25
Knows a place where or a person from whom she would feel comfortable accessing contraception?^b^						
Yes	639	369 (57.8)	1		1	
No	870	396 (45.5)	0.61 (0.50–0.75)		0.65 (0.53–0.81)	
Don’t know	63	35 (55.7)	0.91 (0.54–1.6)	< 0.001	0.95 (0.55–1.6)	< 0.001
Social networks						
Perception that partner supports her using contraception^b^						
Yes	1018	643 (63.2)	1		1	
No	325	101 (31.1)	0.26 (0.20–0.34)		0.27 (0.20–0.35)	
Don’t know	193	44 (22.8)	0.17 (0.12–0.25)	< 0.001	0.17 (0.12–0.25)	< 0.001
Perception that mother supports her using contraception^b^						
Yes	358	195 (54.5)	1		1	
No	710	364 (51.3)	0.88 (0.69–1.1)		0.87 (0.67–1.1)	
Don’t know	341	163 (47.8)	0.77 (0.57–1.0)	0.19	0.76 (0.56–1.0)	0.20
Perception that friends supports her using contraception^b^						
Yes	892	519 (58.2)	1		1	
No	333	43.2 (144)	0.55 (0.43–0.70)		0.54 (0.42–0.70)	
Don’t know	352	40.1 (141)	0.48 (0.37–0.62)	< 0.001	0.48 (0.37–0.63)	< 0.001
Individual knowledge, attitudes and behaviours						
Knowledge about contraception^b^						
0–1	58	22 (37.9)	1		1	
2–3	316	148 (46.8)	1.4 (0.81–2.6)		1.4 (0.74–2.5)	
4–5	1203	634 (52.7)	1.8 (1.0–3.2)	0.03	1.7 (0.95–3.1)	0.08
Misconceptions about contraception						
0–1	494	251 (50.8)	1			
2–3	813	422 (51.9)	(0.83–1.3)			
4	270	131 (48.5)	0.91 (0.66–1.3)	0.64		
Self-efficacy for contraception^b^						
0–1	277	90 (32.5)	1		1	
2–3	608	281 (46.2)	1.8 (1.3–2.4)		1.7 (1.2–2.3)	
4	743	460 (61.9)	3.4 (2.5–4.6)	< 0.001	3.1 (2.3–4.2)	< 0.001
Ever been pregnant						
Yes	385	191 (49.6)	1			
No/don’t know	1352	645 (47.7)	0.93 (0.75–1.1)	0.48		
Ever given birth^b^						
Yes	165	61 (37.0)	1		1	
No	1572	775 (49.3)	1.7 (1.2–2.3)	0.002	1.5 (1.1–2.1)	0.01

P value from design based Wald test

^a^Adjusted ORs: adjusted for age

^b^Adjusted ORs: adjusted for age, highest education level achieved, currently in education, type of area of residence and socioeconomic position

## Discussion

Overall, 45% of sexually active adolescent women in our study reported using MC, a higher proportion than the national prevalence of 22.2% for sexually active, unmarried 15–19 year olds in the 2018 DHS [7]. This is not surprising given that our sample focused on a region of Nigeria with relatively high income, rate of urbanization, and acceptance of use of MC. Contraceptive use may be more acceptable in Ogun state; among married women of reproductive age, 32.1% of women in Ogun use any method of contraception, compared to 16.6% nationally [7].

Available literature documents various beliefs that lead to low uptake of contraception among women of reproductive age, including stigma against premarital sex, unwillingness of parents to discuss contraception with their children, infrequent intercourse, and perception that use of contraception is harmful to health and may hinder a woman’s ability to bear children in the future [22, 26–28]. This stigma also results in exclusion of unmarried adolescents from reproductive health surveys, services and strategies. Studies in both Nigeria and Sierra Leone have shown that sexually active unmarried women have less exposure to family planning information than their married counterparts [27].

Despite holding misconceptions, a majority of our survey respondents had good knowledge about contraception. Our results provided some evidence that this knowledge may be associated with contraceptive use. Similar studies with girls in Ghana have shown no association [22, 26], whilst a study in Tanzania showed higher contraceptive uptake associated with increased knowledge [12]. However, there is extensive evidence from Nigeria that misconceptions about contraception have a negative effect on contraceptive uptake [29, 30].

Respondents reported low levels of social support for use of MC, from partners and from their mothers. Although social support from partners has been shown as important in married women in Nigeria [31–34], there is limited research on partner influence on unmarried girls. However, lack of men’s support for contraceptive use can be a barrier for women [35], and some men believe that their partner’s contraceptive use could lead to promiscuity or infidelity [36, 37]. This highlights the importance of including men in conversations about contraceptive use. Sexual behaviour in unmarried adolescents is taboo for cultural and religious reasons; parents who do not approve of their daughters engaging in sexual activity are not likely to support their contraceptive use. Furthermore, it is often taboo for parents to discuss sexual activity with their children. This limits the ability of girls to learn about MC through interpersonal family channels.

Seventy percent of the respondents using a MC method reported using condoms, with the next most common method being emergency contraception (23%). These findings are in line with the most recent Nigeria DHS, where condoms made up 75% of modern contraceptive use among unmarried sexually active girls [7]. A study conducted in the Ashanti region of Ghana [26] also revealed that condoms (33.3%) were the most used form of contraceptive among sexually active female adolescents. We believe that the common use of condoms, a short-term method of contraception, may be because they are presumed to have no side effects and are more easily available and accessible from providers [38]. This may be particularly important in this population, where although 45% of sexually active girls used contraception, only 39% knew of a place they felt comfortable accessing it—this may limit them to seeking only the most accessible form of contraception. Infrequent sexual activity may also be a reason for a preference for short-term methods. Short-term methods also have little risk of being discovered by parents—relevant for this population where perceived parental approval of contraceptive use is very low.

Many of this survey’s respondents reported using traditional contraceptive methods such as withdrawal or drinking salt solutions (17.8%). The use of traditional contraception is much higher in Nigeria as compared to a similar study in the same demographic group in Tanzania, where only 2% of unmarried adolescent girls aged 15–19 were using a traditional contraceptive method [12]. Although the reasons for this are not clear, data collected from women of reproductive age in Nigeria’s most recent DHS show that women with higher education levels, in higher income quintiles, and in urban areas are more likely to use MC [7]. This implies that mistrust in MC or barriers to accessing MC may be an important factor. A large proportion of unmarried girls who reported having ever been pregnant also reported that they had never given birth which suggests that some of the women interviewed may have terminated their pregnancies emphasising the high unmet need in this population.

In contrast to the high rates of short-term contraceptive use, there is a large global emphasis on encouraging long-acting reversible contraceptives (LARCs). These methods have advantages—they are female-controlled—but they also have disadvantages—they may face significant access barriers, can be more likely to cause side effects, and do not protect against sexually transmitted infections. Girls who have sex infrequently, such as those in our survey population, may also feel that the infrequent protection offered by condoms is sufficient; they may also feel that the risk of side effects from LARCs is too high given their infrequent sexual activity.

Injectable contraceptive use was very low in this population, with less than 1% of sexually active girls reporting use of this method. Although adolescents in sub-Saharan Africa have been reported to prefer injectable contraceptive methods, these methods are less popular in Nigeria [39]. The 2018 DHS reports that 3% of married women aged 15–49 and 2% of unmarried women aged 15–49 currently using a contraceptive method are using an injectable method. Although injectables are more commonly used in Nigeria’s South West, they are only used by 0.6% of women aged 15–49 in Ogun state.

### Strengths

This survey targeted sexually active unmarried adolescents aged 15–19, a group rarely covered in family planning literature despite the unique barriers they face in accessing contraception. Most of the data available on determinants of contraceptive use are based on samples of married adult women [40] or are representative of all women aged 15–49 [41]. Even where girls are included, data is often presented for the 15–24 year old age group, and absolute numbers of adolescent women surveyed are quite small. For example, the 2018 Nigeria DHS surveyed only 310 sexually active unmarried women aged 15–19 across the entire nation [7].

This survey targeted a large number of unmarried adolescent girls in Ogun state, Nigeria. The sampling method makes this data largely generalizable for the LGAs where the study was conducted. Ogun state is a large, highly populated and largely urban state with similar cultural context to other neighbouring states; this study could therefore inform programming for a large population of adolescent girls living in urban areas in Nigeria. Furthermore, these findings could be used to inform future research and programming in urban adolescents across Africa as the continent’s urban populations continue to increase [42].

### Limitations

The findings of this survey cannot describe causality of use of MC, a weakness of all cross-sectional analyses. It is unclear, for example, whether women use MC because they are knowledgeable about it, or women are knowledgeable about MC because they are using it. Similarly, it is not clear that reaching higher education makes women more likely to use MC—women using MC may be more likely to reach higher education because they are not shut out of the school system due to early pregnancy.

The data collection relied on self-reporting of sensitive information. Despite the best efforts of the research team to minimize risks there is potential misreporting of information from survey participants [7].

This survey reached only girls residing in households at the time of interview; the non-response rate was largely due to girls being away at school, which could limit generalizability of the results to some extent.

Finally, the data and conclusions may be affected by unmeasured confounding, and conclusions may not be fully representative due to chance.

### Recommendations

Reproductive health programming tailored to the needs of unmarried sexually active adolescent women in South Western Nigeria should seek to increase access to modern contraceptives and promote accurate information about traditional methods with no known efficacy, e.g. drinking salt solutions.

Emphasis on increasing social acceptance of contraceptive use, especially by parents, should also be a priority. Finally, two of the more common contraceptive methods women reported using—condoms and withdrawal—rely on male control. Programmes promoting contraceptive use should also incorporate female-controlled options for birth control and should also engage men as key participants in reproductive health.

Programmes aiming to increase voluntary uptake of MC among unmarried adolescent girls should also incorporate activities to combat the stigma of girls accessing contraception and should seek to identify and remove barriers that young women face in accessing modern methods.

## Conclusions

This study demonstrates that unmarried adolescent women in South West Nigeria are sexually active and have unmet need for family planning. Although most unmarried girls have heard of modern contraception, the majority do not know a place they would feel comfortable accessing it. Programming that seeks to increase unmarried adolescents’ access to modern contraception should promote accurate information about modern contraception, provide multiple options including long-term and short-term contraceptive options, and should engage male partners of girls as well. Supporting girls in delaying childbearing until they desire a child would not only reduce the risks of early pregnancy but would also support girls in completing school and achieving their goals for adult life.

## Data Availability

The datasets used during the current study are available from the corresponding author on reasonable request.

## Abbreviations

A360: Adolescent 360
LGA: Local government areas
MC: Modern contraception
SSA: Sub-Saharan Africa
SDG: Sustainable Development Goals
FP2020: Family planning 2020
EA: Enumeration area
DHS: Demographic and Health Survey
CAPI: Computer-Assisted Personal Interviewing
mCPR: Modern Contraceptive Prevalence Rate
LARC: Long-acting reversible contraceptives
IUCD: Intrauterine contraceptive device
SDM: Standard days method
LAM: Lactational amenorrhoea method
